# Altered Functional Connectivity of Insular Subregions in Alzheimer’s Disease

**DOI:** 10.3389/fnagi.2018.00107

**Published:** 2018-04-11

**Authors:** Xingyun Liu, Xiaodan Chen, Weimin Zheng, Mingrui Xia, Ying Han, Haiqing Song, Kuncheng Li, Yong He, Zhiqun Wang

**Affiliations:** ^1^Department of Radiology, Dongfang Hospital, Beijing University of Chinese Medicine, Beijing, China; ^2^State Key Laboratory of Cognitive Neuroscience and Learning, Beijing Normal University, Beijing, China; ^3^Beijing Key Laboratory of Brain Imaging and Connectomics, Beijing Normal University, Beijing, China; ^4^International Data Group (IDG)/McGovern Institute for Brain Research, Beijing Normal University, Beijing, China; ^5^Department of Neurology, Xuanwu Hospital, Capital Medical University, Beijing, China; ^6^Department of Radiology, Xuanwu Hospital, Capital Medical University, Beijing, China; ^7^Beijing Key Laboratory of Magnetic Resonance Imaging and Brain Informatics, Beijing, China

**Keywords:** insula, network, functional connectivity, fMRI, Alzheimer’s disease

## Abstract

Recent researches have demonstrated that the insula is the crucial hub of the human brain networks and most vulnerable region of Alzheimer’s disease (AD). However, little is known about the changes of functional connectivity of insular subregions in the AD patients. In this study, we collected resting-state functional magnetic resonance imaging (fMRI) data including 32 AD patients and 38 healthy controls (HCs). By defining three subregions of insula, we mapped whole-brain resting-state functional connectivity (RSFC) and identified several distinct RSFC patterns of the insular subregions: For positive connectivity, three cognitive-related RSFC patterns were identified within insula that suggest anterior-to-posterior functional subdivisions: (1) an dorsal anterior zone of the insula that exhibits RSFC with executive control network (ECN); (2) a ventral anterior zone of insula, exhibits functional connectivity with the salience network (SN); (3) a posterior zone along the insula exhibits functional connectivity with the sensorimotor network (SMN). In addition, we found significant negative connectivities between the each insular subregion and several special default mode network (DMN) regions. Compared with controls, the AD patients demonstrated distinct disruption of positive RSFCs in the different network (ECN and SMN), suggesting the impairment of the functional integrity. There were no differences of the positive RSFCs in the SN between the two groups. On the other hand, several DMN regions showed increased negative RSFCs to the sub-region of insula in the AD patients, indicating compensatory plasticity. Furthermore, these abnormal insular subregions RSFCs are closely correlated with cognitive performances in the AD patients. Our findings suggested that different insular subregions presented distinct RSFC patterns with various functional networks, which are differently affected in the AD patients.

## Introduction

Alzheimer’s disease (AD) is a progressive neurodegenerative disease characterized by memory and other cognitive decline. Pathologically, AD presented the deposition of amyloid-β plaques, neurofibrillary tangles, and neuronal loss (Braak and Braak, 1991), which disrupted the specific brain network and might contribute to memory and cognitive decline (Buckner et al., 2009; Seeley et al., 2009; Pievani et al., 2011).

Resting-state functional magnetic resonance imaging (rs-fMRI) has proven to be a highly effective method for analyzing complex neural networks by measuring the intrinsic brain fluctuations in the blood-oxygen level-dependent (BOLD) signals (Biswal et al., 1995; Zhang and Raichle, 2010). Many rs-fMRI studies in AD have revealed disrupted resting state functional connectivities (RSFCs) in the default mode network (DMN) regions, including the posterior cingulate cortex (PCC) (Greicius et al., 2004; Zhang et al., 2010), hippocampus (Wang et al., 2006; Allen et al., 2007), inferior parietal lobe (IPL) (Wang et al., 2015), prefrontal cortex (Wang et al., 2007; Dai et al., 2012), and thalamus (Wang et al., 2012). These regions constituted the task negative network, which showed deactivation during the cognitively demanding tasks. However, recent studies have suggested the existence of other networks which are associated with AD (Seeley et al., 2007; Palesi et al., 2016), For example, the executive control network (ECN), covering several medial and lateral prefrontal cortex, temporal and parietal regions; the salience network (SN); which encompasses the anterior cingulate cortex (ACC) and the frontoinsular circuit; sensorimotor network (SMN) including precentral and postcentral cortex. These above networks showed increased RSFCs during the performance of demanding tasks, which were defined as the task-positive networks (Fox et al., 2005). Previous fMRI studies have found disrupted RSFCs within the ECN, SN, SMN as well as other task-positive networks in the AD patients (Agosta et al., 2012; Brier et al., 2012; Wang et al., 2015). However, recent rs-fMRI study found that there were anti-correlation between the task positive and negative networks. In addition, the study found the AD patients presented disrupted DMN connectivity while enhanced SN connectivity (Zhou et al., 2010). As we known, task positive networks (ECN, SN, and SMN) were involved in externally oriented attention and exhibited important functions in performing tasks, while, task negative network such as DMN was implicated in internally oriented or self-related cognition. We speculated there might be a core region mediating the task negative and positive networks and providing an interface between internal motivational states and external information.

Recent studies provided the possible answer that the insula (part of SN) played a critical role in the dynamic switching between the task positive networks (ECN and SN) and negative network (DMN) (Sridharan et al., 2008; Menon and Uddin, 2010; Liang et al., 2016). As a critical hub of the brain network, the insula is anatomically associated with the cortical, limbic, and paralimbic structures. It is functionally related to the higher-order cognition, autonomic, emotion, and sensory processes (Naqvi and Bechara, 2010). Previous magnetic resonance imaging (MRI) studies have demonstrated insular gray matter (GM) loss (Guo et al., 2012), abnormal insular activities (Lin et al., 2017), and disrupted insular network in the early stage of AD (Xie et al., 2012). These studies raised the interest of insula, which may be the most vulnerable region and core integral hub in the AD patients.

However, the insula is composed of several subregions which involves in multimodal functions such as cognitive, sensorimotor processing and so on. Several recent studies mapped the organization of the human insula. By using diffusion tensor imaging (DTI)-based tractography, Nanetti et al. (2009) divided the left insula into anterior and posterior subregions based on connectivity information. Previous research used different locations in the insula as seeds to invest whole-brain RSFC patterns and found that anterior insula is associated with dorsal anterior cingulate, while posterior insula (PI) is mainly connected with SMA and middle cingulate (Taylor et al., 2009). By using a data-driven clustering technique, recent studies subdivided the insula into three part based on whole-brain RSFC maps, and found that there were functionally connection between the PI and somatomotor cortices; the dorsal anterior insula (dAI) and dorsal ACC along with other regions; as well as the ventral anterior insula (vAI) and pregenual ACC (Deen et al., 2011; Zhang et al., 2016).

Considering the insula heterogeneity and its vulnerable characteristics in AD, it is extremely critical to reveal the intrinsic functional connectivity patterns of the insular subregions in AD. However, few previous rs-fMRI studies paid attention to insular-connectivity or SN changes in the early AD patients (Xie et al., 2012; He et al., 2014). One study employed independent component analysis (ICA) to analyze the SN alteration in the AD patients. Although ICA could use statistical method to decompose BOLD data into independent components, however, there are some problems for the ICA method to be resolved. For example, there are no priori criteria on how many independent components should be identified in BOLD data, which may influence the final results in a high degree. Another rs-fMRI study explored functional connectivity in the early stage of AD. However, the study only selected four insular subdivisions as seed regions to detect the insular connectivity on mild cognitive impairment. Moreover, the BOLD data analysis used 1.5T MRI device which might produce different results from 3.0T. At present, no studies have reported patterns of intrinsic insular subregional connectivity in the AD patients using 3.0T MRI.

In this study, we aim to explore the intrinsic functional connectivity of the insular subregions in the AD patients by using rs-fMRI. We plan to identify three subregions of the each side insula and calculate the whole-brain RSFCs of each subregion, which was followed by a group comparison. We strove to determine whether the AD patients’ exhibit differentially disrupted functional connectivity patterns of insular subregions. We hypothesized that several cognitive-related RSFC patterns were identified from anterior to posterior regions of insula, which included SN, ECN, and SMN, as well as the negative connectivity network (DMN). When comparing the RSFC patterns between the two groups, the AD patients presented special insular patterns including disrupted connectivity in some networks while enhanced connectivity in other networks. Furthermore, insular connectivity changes are closely associate with the clinical behavioral scores in the AD patients.

## Materials and Methods

### Participants

All the subjects recruited for this study included 34 patients with AD and 41 healthy controls (HCs). The AD patients were collected from memory clinic at Xuanwu Hospital. The diagnosis of AD patients was confirmed using the new research criteria for possible or probable AD (Dubois et al., 2007, 2010). Complete physical, neurological, and neuropsychological assessments were performed on all the AD subjects including mini-mental state examination (MMSE), auditory verbal learning test (AVLT), the extended scale for dementia (ESD), montreal cognitive assessment (MoCA), clock drawing task (CDT), Activity of Daily Living Scale (ADL), functional activities questionnaire (FAQ), Hamilton Depression Scale (HAMD), Hachinski Ischemic Score (HIS), and clinical dementia rating (CDR) score (Morris, 1993). The HCs were recruited from the local community by advertisements. The inclusion criteria for HCs were as follows: (1) no neurological or psychiatric disorders and no neurological deficiencies; (2) no abnormal findings, in conventional brain MRI; (3) no cognitive complaints; and (4) MMSE score of 28 or higher and CDR score of 0. After excluding five subjects (two AD patients and three HCs) in the Image preprocessing, 32 AD patients and 38 HCs were included in this study finally. The clinical data for the remaining 70 participants were shown in **Table 1**. This study was carried out in accordance with the recommendations of State Key Laboratory of Cognitive Neuroscience and Learning of Beijing Normal University and Medical Research Ethics Committee of Xuanwu Hospital. The protocol was approved by the Medical Research Ethics Committee of Xuanwu Hospital. All subjects gave written informed consent in accordance with the Declaration of Helsinki.

**Table 1:** Demographic and neuropsychological test.

	AD (*n* = 32)	HC (*n* = 38)	*P*-Value
Age (years)	52–86 (71.25 ± 8.63)	50–86 (68.39 ± 7.78)	0.15^a^
Gender (male/female)	14/18	13/25	0.41^b^
CDR	0.5 (*n* = 14), 1 (*n* = 18)	0	–
MMSE	10–25 (18.56 ± 3.99)	28–30 (28.63 ± 0.67)	<0.001^a^
AVLT	8–24 (14.81 ± 4.12)	39–52 (44.42 ± 2.74)	<0.001^a^
ESD	107–200 (155.33 ± 26.48)	180–248 (227.74 ± 15.68)	<0.001^a^
MoCA	8–19 (14.94 ± 3.23)	27–30 (28.63 ± 0.67)	<0.001^a^
CDT	3–8 (6.13 ± 1.43)	8–9 (8.71 ± 0.46)	<0.001^a^
ADL	22–45 (30.41 ± 7.21)	20–22 (21.08 ± 0.78)	<0.001^a^
FAQ	4–11 (6.25 ± 1.70)	0–2 (0.55 ± 0.76)	<0.001^a^
HAMD	0–3 (1.06 ± 1.08)	0–3 (0.61 ± 1.00)	0.07^a^
HIS	0–3 (1.16 ± 0.77)	0–3 (1.13 ± 1.07)	0.91^a^


*Data are presented as the range of minimum–maximum (mean ± SD). AD, Alzheimer’s disease; ADL, Activity of Daily Living Scale; AVLT, World Health Organization–University of California–Los Angeles Aauditory verbal learning test; CDR, clinical dementia rating; CDT, clock drawing task; ESD, extended scale for dementia; FAQ, functional activities questionnaire; HAMD, Hamilton Depression Scale; HC, healthy controls; HIS, Hachinski Ischemic Score; MMSE, mini-mental state examination; MoCA, montreal cognitive assessment; AD, Alzheimer’s disease; HC, healthy controls. ^*a*^The P-value was obtained by two-sample two-tailed t-test; ^*b*^The P-value was obtained by two-tailed Pearson chi-square test.*

### Data Acquisition

Magnetic resonance imaging was performed on a SIEMENS Trio 3-Tesla scanner (Siemens; Erlangen, Germany). Foam padding and headphones were used to limit head motion and reduce scanner noise separately. The rs-fMRI data were acquired axially using an echo-planar imaging (EPI) sequence: Repetition time (TR)/echo time (TE)/flip angle (FA) = 2000 ms/40 ms/90°, field of view = 24 cm × 24 cm, resolution = 64 × 64 matrix, slices = 28, thickness = 4 mm, gap = 1 mm, voxel size = 3.75 mm × 3.75 mm × 4 mm, and bandwidth = 2232 Hz/pixel. 3D magnetization-prepared rapid gradient echo (MPRAGE) T1-weighted sequence: TR/TE/inversion time (TI)/FA = 1900 ms/2.2 ms/900 ms/9°, resolution = 256 × 256 matrix, slices = 176, thickness = 1 mm, voxel size = 1 mm × 1 mm × 1 mm. During scans, subjects were instructed to hold still, keep their eyes closed but not to fall asleep, and not to think of anything in particular, and not to fall asleep.

### Data Preprocessing

The preprocessing of the rs-fMRI data was performed using the Statistical Parametric Mapping software (SPM8^1^) toolkits. Briefly, preprocessing included the first 10 volumes removal, slice timing correction and head motion correction. Then, the T1-weighted images were applied to coregister the functional image to their corresponding anatomical image, and the resulting aligned T1 dataset was registered into Montreal Neurological Institute (MNI) space. To reducing the inaccuracy of the coregistration of the fMRI data due to GM atrophy in AD patients and HCs, a custom T1 template was created by averaging the normalized anatomical images across all subjects. Then, the normalized functional images were generated by applying the transformation of the T1 images to the customized T1 template. Functional images were resampled to 3 mm isotropic voxels and spatially smoothed with a 4 mm full width-half maximum (FWHM) Gaussian kernel and removal of linear trends. Temporal band-pass filtering (0.01–0.1 Hz) were applied to reduce the effect of low-frequency drifts and high-frequency physiological noise. Finally, several nuisance variables, including twenty-four head motion parameters, cerebrospinal fluid signal, white matter signal, and global mean signal were regressed by multiple linear regression analysis. During image preprocessing, five subjects (two AD patients and three HCs) were excluded because of failures in image normalization.

### Definition of Insular Subregions

**FIGURE 1:**
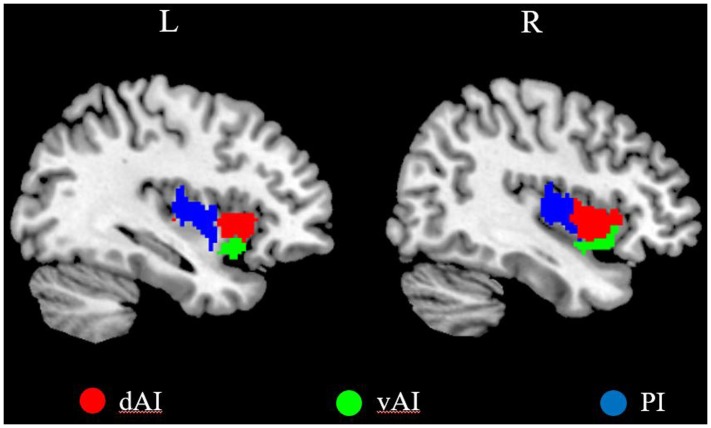
The left and right insula subregions including dorsal anterior insula [dorsal anterior insula (dAI), red], ventral anterior insula (vAI, green), and posterior insula (PI, blue).

We defined three insular subregions in each hemisphere according to a previous connection-based parcellation study (Deen et al., 2011), in which the insular lobe was subdivided based on whole brain functional connectivity, through data-driven clustering technique. The cluster analysis identified 3 insular subregions in each hemisphere: vAI, dAI, and PI (Deen et al., 2011), as shown in **Figure 1**.

### RSFCs Analysis of the Insular Subregions

For each subject, we calculated the Pearson’s correlation coefficients between the mean time series of each voxel of the insular subregion and the time series of all voxels in the other gray matter and then converted to *z*-values using Fisher’s r-to-z transformation to improve the normality. Finally, for each subject, we generated six *z*-score maps that represented the intrinsic RSFC patterns of the six insular subregions.

### Statistical Analysis

For each group, individuals’ *z*-values were entered into a random-effect one-sample *t*-test in a voxel wise manner to detect brain regions that presented significant positive or negative correlations with each insular subregion. Then we extracted the positive and negative RSFC maps of each insular subregion in both groups and merged these positive and negative RSFC maps of each insular subregion, respectively, as the masks for the subsequent analysis. Then, to compare the intergroup differences in insular RSFC patterns after controlling for the age, gender and education, we performed two-sample *t*-test within the positive and negative RSFC masks, respectively. These analyses were false discovery rate (FDR) corrected for multiple comparisons (*p* < 0.05, two tailed).

To test the relationship between the RSFCs of each insular subregion with significant intergroup differences and the cognitive behaviors, we calculated the Spearman’s correlation coefficients between these RSFCs and the cognitive behavior scores, with age, gender and education as covariates. The statistical significance level was set at *p* < 0.05.

## Results

### Demographic and Neuropsychological Tests

Demographic characteristics were shown in **Table 1**. No significant differences of gender and age were found between the AD and HC groups (both *P*s > 0.01). However, the MMSE, AVLT, ESD, MoCA, CDT, ADL, and FAQ scores showed significantly lower in the AD group than that of the HC group (*P*s < 0.0001).

### Within-Group RSFCs of the Insular Subregions

**FIGURE 2:**
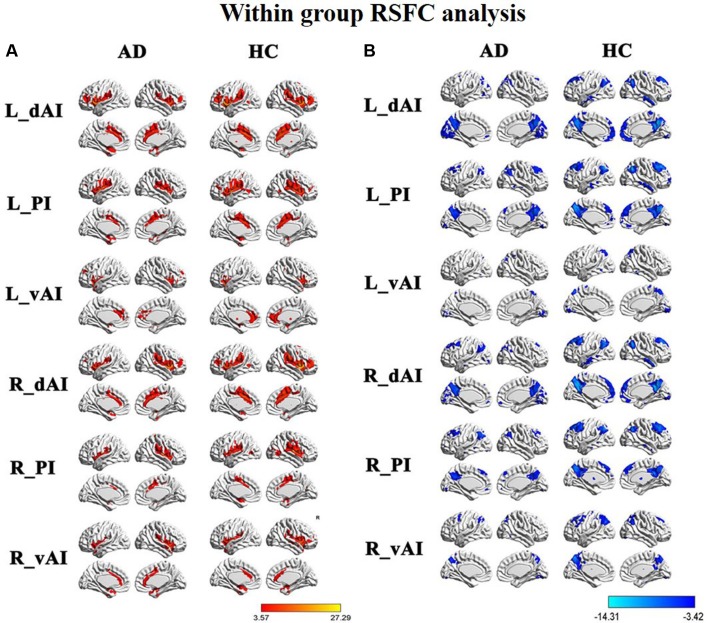
Within group resting-state functional connectivity (RSFC) analysis, **(A)** The positive RSFC patterns of the insular subregions in each group including healthy controls (HCs) and *Alzheimer’s disease* (AD) patients [*P* < 0.05, false discovery rate (FDR) corrected]; **(B)** the negative RSFC patterns of the insular subregions in each group including healthy controls and AD patients [*P* < 0.05, FDR corrected].

**Figure 2** illustrated the functional connectivity maps for each insular subregion within the HC and AD groups. By visual inspection, within group connectivity map of the HC and AD exhibited similar patterns. We identified three insular subregions by each side and mapped the similar whole-brain functional connectivity patterns between the two sides. For example, in the left side of HC group, we found that the dAI exhibited positive connectivity with the ECN regions, mainly including the bilateral insula, middle temporal gyrus (MTG), dorsal lateral prefrontal cortex (DLPFC), inferior frontal gyrus (IFG), IPL, ACC, and middle cingulate cortex (MCC). Additionally, negative connectivity was involved in the DMN regions, including the precuneus, PCC, medial prefrontal cortex (MPFC), IPL, MTG, and inferior temporal gyrus (ITG).

The vAI was positively associated with the SN regions, involving the bilateral insula and pregenual ACC, MPFC, as well as a small cluster in the IPL. Negative connectivity was primarily showed in the middle frontal gyrus (MFG), precuneus, IPL, and middle occipital gyrus (MOG), most of which are involved in DMN.

The posterior zone of the insula (PI) was typically regarded as a component of the SMN. Positive connectivity was showed with the bilateral insula, temporal lobe and parietal cortices, as well as MCC. Notably, we observed that the PI showed significant negative connectivity with the MPFC, PCC, precuneus, IPL, and MTG, which constituted the classical pattern of the DMN.

### Between-Group RSFC Differences of the Insular Subregions

**FIGURE 3:**
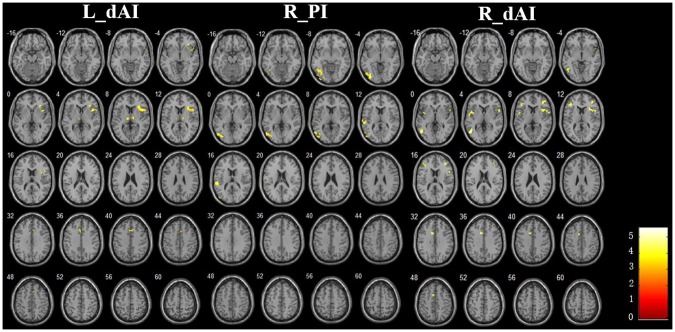
The decreased positive RSFCs of insular subregions in AD patients (*P* < 0.05, FDR corrected).

The **Figure 3** and **Table 2** illustrated the between-group differences of the positive functional connectivity for each insula subregion. When comparing the functional connectivity of the dAI between the AD and HC groups, significantly decreased positive RSFCs were revealed in the AD patients in several regions predicting to the ECN regions. The detailed regions were as follows: For the left dAI, we observed decreased positive functional connectivity mainly in the right IFG, left thalamus and MCC in the AD patients. For the right dAI, we found the decreased positive functional connectivity mainly in the bilateral insula, left MTG, right MFG, left IFG, and left MCC.

**Table 2:** Regions showing decreased positive AD-related RSFCs in insular subregions.

ROIs	Brain regions	Cluster voxels	MNI Coordinates (mm)			Maximum Z
				
			*x*	*Y*	*z*	
L. dAI	R.IFG	140	45	15	6	-5.52
	L.Thalamus	41	0	-15	6	-4.38
	R.MCC	35	9	21	36	-3.90
R. dAI	R.Insula	73	33	15	12	-4.39
	L.MTG	64	-48	-63	3	-4.79
	L.Insula	39	-42	3	3	-4.14
	R.MFG	31	39	39	12	-4.61
	L.IFG	31	-42	30	9	-3.91
	L.MCC	33	-9	12	36	-4.19
R. PI	R.IOG	216	-36	-75	-3	-4.87
	L.STG	57	-54	-30	15	-5.57


*Between groups differences were determined by two-sample t-tests [p < 0.05, false discovery rate (FDR) corrected, two tailed]. IFG, inferior frontal gyrus; MCC, middle cingulate cortex; MTG, middle temporal gyrus; MFG, middle frontal gyrus; IOG, inferior occipital gyrus; STG, superior temporal gyrus; L, left; R right; RSFC, resting-state functional connectivity; AD, Alzheimer’s disease; MNI, Montreal Neurological Institute; dAI, dorsal anterior insula; PI, posterior insula.*

Compared with the HCs, the AD patients exhibited decreased positive functional connectivity with the right PI in several regions including the left inferior occipital gyrus (IOG) and superior temporal gyrus (STG). We didn’t observe differences of the positive functional connectivity for the left PI and bilateral vAI between the AD and HC groups.

**FIGURE 4:**
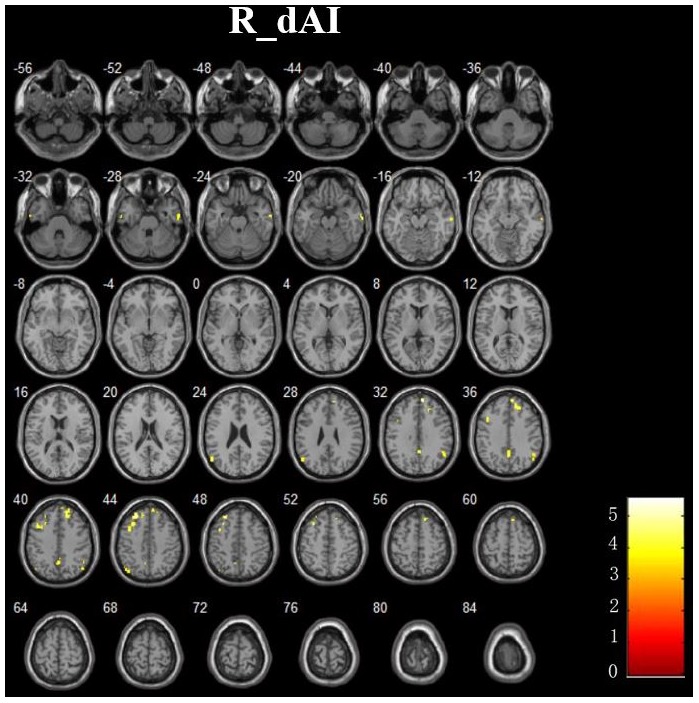
The increased negative RSFCs of insular subregions in AD patients (*P* < 0.05, FDR corrected).

In addition, **Figure 4** and **Table 3** showed the between-group differences of the negative functional connectivity for each insula subregion. For the right dAI, we found that the AD group showed significantly increased negative functional connectivity with the DMN regions, including the left ITG, right MTG, right MPFC, bilateral angular gyrus, right precuneus, and left MFG. No other differences of the negative functional connectivity were found between the two groups.

**Table 3:** Regions showing increased negative AD-related RSFCs in insular subregions.

ROIs	Brain regions	Cluster voxels	MNI coordinates (mm)			Maximum Z
				
			*x*	*y*	*z*	
R. dAI	L.ITG	30	-57	-12	-30	3.90
	R.MTG	45	66	-9	-24	4.36
	R.MPFC	163	9	54	33	4.85
	L. Angular gyrus	39	-54	-66	24	3.90
	R. Angular gyrus	46	54	-54	33	3.82
	R.Precuneus	71	3	-57	36	3.91
	L.MFG	186	-27	36	45	5.14


*Between groups differences were determined by two-sample t-tests [p < 0.05, false discovery rate (FDR) corrected, two tailed]. ITG, inferior temporal gyrus; MTG, middle temporal gyrus; MPFC, medial prefrontal cortex; MFG, middle frontal gyrus; L, left; R right; RSFC, resting-state functional connectivity; AD, Alzheimer’s disease; MNI, Montreal Neurological Institute; dAI, dorsal anterior insula.*

To show the relationship among the insular subregions and other brain networks, here, we mainly focused the result of removing the global signal, but as reference to compare, we also performed the preprocessing again without removing the global signal and the results are shown in the Supplementary Materials (see **Supplementary Table S1** and **Supplementary Figures S1**–**S3**). As the result, the significantly decreased positive RSFCs were found in the AD patients in several regions: For the left dAI, we observed decreased positive RSFCs mainly in the right IFG, right lingual gyrus, left thalamus and left MTG in the AD patients. For the right dAI, we found decreased positive RSFCs in the left MOG, right superior occipital gyrus (SOG), left MTG, and right IFG. For the left PI, the AD patients exhibited decreased positive functional connectivity with the right MOG and left MTG, while for the right PI, the region only included MOG. For the left vAI, we found the decreased RSFCs mainly in the right IFG and caudate. However, we didn’t find the differences of RSFCs in the right vAI between the two groups.

### Relationship Between the Insular Subregional RSFCs and Cognitive Performances

**FIGURE 5:**
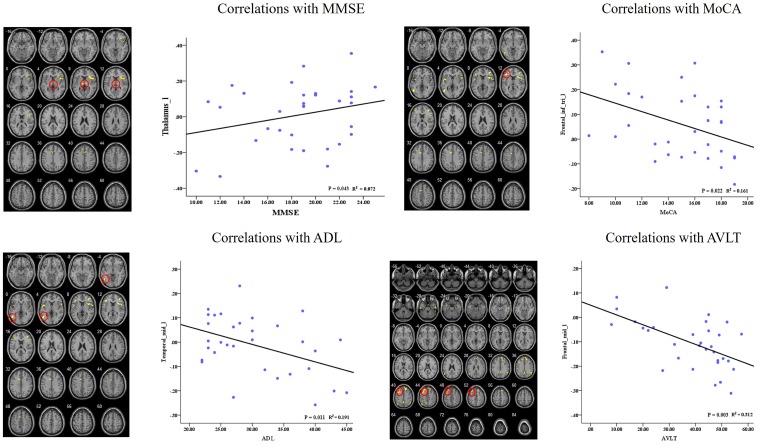
Correlation of clinical variables and resting-state functional connectivity of insular subregions.

In the AD group, we showed significant positive correlations between the MMSE scores and the RSFC of the left dAI and left thalamus. Besides, we also found significant negative correlations between cognitive behavior scores (i.e., MoCA, ADL, and AVLT) and the RSFCs of right dAI and several regions (i.e., IFG, MTG, and MFG). Please see **Figure 5** for detail.

## Discussion

In the current study, by analysis of resting-state fMRI data acquired from both HC and AD patients, we demonstrated distinctive patterns of RSFC in each subregion of insula, which are supported by recent anatomical studies. For positive connectivity, three cognitive-related RSFC patterns were identified within insula that suggest anterior-to-posterior functional subdivisions: (1) an dorsal anterior zone of the insula that exhibits RSFC with insula, MTG, DLPFC, IFG, IPL, ACC, and MCC, which involved in ECN; (2) a ventral anterior zone of insula, exhibits functional connectivity with the bilateral insula, pregenual ACC and MPFC, which involved in the SN; (3) a posterior zone along the insula exhibits functional connectivity with bilateral insula, temporal lobe and parietal cortices, as well as MCC, most of which is the component of the SMN. In addition, we found significant negative connectivities between the each insular subregion and several special regions, including the MPFC, PCC, precuneus, IPL, MTG, and so on, which constituted the classical pattern of the DMN.

Comparison between the AD patients and HCs revealed the following findings. First, AD patients showed the disruption of positive RSFCs in the different network (ECN and SMN). There were no differences of the positive RSFCs in the SN between the two groups. Second, on the other hand, some DMN regions showed increased negative RSFCs to the sub-region of insula in the AD patients relative to HCs. Finally, we found that insular connectivity changes were closely correlated with cognitive performances in AD patients.

### Within Group Insular RSFCs Including ECN, SN, SMN, and DMN

In the previous studies, ECN has been identified in the insular connectivity analysis, which includes DLPFC, presupplementary motor area, IFG, posterior parietal and premotor cortices and so on (Damoiseaux et al., 2006; Seeley et al., 2007; Uddin et al., 2010). Among these regions, DLPFC and parietal neocortices were consistently reported, which were involved in cognitive control during task performance. In our study, taking insular-subregion (dAI) as seed region, we defined the network of ECN, which is similar with the previous studies.

The nervous system is continuously activated by internal and extra-personal stimuli. Several previous studies identified the SN in humans, which showed consistent activation during emotionally internal and external stimuli (Damoiseaux et al., 2006; Seeley et al., 2007; Uddin et al., 2011). The network consists of paralimbic structures, most prominently the ACC and orbital frontal insula, extending to the basal ganglia and striatum, which is responsible for guidance of thought and behavior (Seeley et al., 2007; Uddin et al., 2010). In our study, within group connectivity analysis showed the positive connectivity between the bilateral vAI and pregenual ACC, medial frontal cortex, IPL, which were overlapped with the SN regions.

For the PI connectivity analysis, we detected the associated regions of bilateral insula, temporal lobe and parietal cortices, which suggested the SMN. This network had connectivity with primary sensory and motor cortices indicating the important role of the related function (Beckmann et al., 2005; De Luca et al., 2006; van den Heuvel et al., 2008).

Of special interest, is so called DMN, consisting of functionally linked regions such as PCC, MPFC, IPL, and temporal lobe (Raichle et al., 2001; Buckner et al., 2008). The activity and connectivity of DMN played important role in the cognitive and emotional processing (Greicius et al., 2003), external world monitoring (Gusnard et al., 2001), and mind wandering (Mason et al., 2007). From the within-group insula connectivity analysis, we noticed most of the regions negatively connecting to insula were overlapped with DMN regions. A previous study reported the insula was inversely correlated with the PCC, a crucial region within the DMN (Greicius et al., 2003). In addition, some previous studies reported the relationship among the insular networks, which revealed the critical role of the insula (core node of SN) in switching between the ECN and the DMN. These regions interact competitively during cognitive information integration, once a salient stimulus is found, the orbital frontal insula of SN transfer the appropriate transient control signals to utilize the ECN to regulate higher-order cognitive processes such as attention and working memory, while not utilize the DMN regions (Fox et al., 2005; Sridharan et al., 2008; Menon and Uddin, 2010). Our result showed the positive correlation of SN, ECN and negative correlation of DMN in the insular connectivity analysis, which is consistent with the previous study.

### Between Group Differences of Insular RSFCs

When comparing the RSFCs of insula between the two groups, the decreased positive RSFCs in the AD patients was found in several special regions predicting to the ECN and SMN, while we did not find the disruption of SN regions in the AD patients relative to HCs. For the disconnection of ECN regions, we speculate that the control deficits of the AD patients can be explained by the dysfunction of the ECN. In the previous task-related studies, reduced PET activation was found in the right frontal cortex and MTG in the AD patients (Johannsen et al., 1999) and also reduced fMRI activation in the frontal regions was revealed in the AD patients (Hao et al., 2005). Resting state-fMRI studies have confirmed disrupted RSFCs within the ECN regions in the AD patients (Liang et al., 2011; Brier et al., 2012; Wang et al., 2015). The findings are consistent with some previous studies, which also showed the disruption of the ECN.

Between groups analysis revealed decreased connectivity in some SMN regions in the AD patients. In previous study, decreased activation of the supplementary motor area and premotor cortex has been found in AD by using motor-related tasks (Agosta et al., 2010; Vidoni et al., 2012). By analysis of 510 human data, a resting state-fMRI study revealed the preferentially affected SMN in the AD patients (Brier et al., 2012). Together with the previous findings, we speculated that there might be subtle impaired SMN in the AD patients.

However, we didn’t find the differences of the SN between AD and HC group. Previous literatures showed inconsistent results about SN changes of AD. For example, two previous studies found increased functional connectivity of SN in the AD patients (Zhou et al., 2010; Agosta et al., 2012), while another study found the disruption of SN in the early stage of AD (Liang et al., 2012). Recent study found the disrupted structural and functional organizations of the SN in MCI and AD patients (He et al., 2014). In our study, we did not detect the alteration of SN in the AD patients. We speculated there are several reasons: first, the samples are different among these studies; second, the data analysis methods are not completely identical, for example, some studies use the ICA methods while other studies selected different region of interest to explore the RSFC patterns of the disease. Further studies need to bedone in the future.

In between group analysis, the AD patients demonstrated increased negative connectivity between the dAI and several DMN regions. Altered connectivity of the DMN may have implications for cognitive function. In the previous fMRI studies of AD, parietal over-activation was detected by using a face-name recognition task (Pariente et al., 2005). Increased PCC activation was found in individuals at high risk for AD during a semantic memory task (Seidenberg et al., 2009). A previous resting state fMRI study reported the increased RSFCs of part DMN regions (IPL and PCC) in the AD patients, which suggested functional recruitment of these regions in AD (Wang et al., 2015). Previous study revealed enhanced connectivity in the ventral and anterior DMN by using ICA (Damoiseaux et al., 2012). By using seed-based RSFCs analysis, a recent study demonstrated enhanced functional connectivity among the PCC, the parahippocampus, and the anterior hippocampus in the early stage of AD, which suggests a maladaptive mechanism (Gardini et al., 2015).

### Correlation Between Amyloid-β Deposition and Insular RSFCs

The AD pathological changes including the Aβ plaques, neurofibrillary tangles and significant volume atrophy have been consistently demonstrated in the insula in previous studies (Braak et al., 2006; He et al., 2015; Zhao et al., 2015). Furthermore, recent studies revealed that cognitive performance was closely associated with the RSFCs of the insula in MCI patients, which is the early stage of AD (Xie et al., 2012; Nellessen et al., 2015). Based on these two main findings, we speculated that the AD pathological changes deposited on the insula or related regions, disrupted the insular activity and connectivity, finally resulted in the disruption of the insular related networks including ECN and SMN, which caused the cognitive control and sensory motor deficit. Furthermore, due to the dynamic balance between the insular anticorrelate networks, The DMN regions showed increased connectivity to resist the disruption of ECN and SMN.

As we known, the amyloid-β deposition is the primary pathological changes of the AD. Many studies have discussed the relationship between amyloid-β deposition and functional brain connectivity. Several studies reported reduced RSFCs of the DMN in cognitively normal subjects with brain amyloid burden. However, a recent study analyzed the influences of amyloid-β deposition on the three large-scale networks (DMN, SN, and ECN) in older adults with normal cognition (Lim et al., 2014). Compared with the Pittsburgh compound B (PIB) negative group, the increased DMN, decreased ECN as well as not different of SN were found in the PIB positive group. Interestingly, the results were very similar with our findings on the subject of AD, which is different from the previous studies that presented lower DMN functional connectivities in AD, MCI and cognitively normal subjects with amyloid burden (Sheline et al., 2010; Agosta et al., 2012). In this recent study, the discrepancy in DMN was explained as due to the “acceleration” and “brain reserve” hypotheses. The ‘acceleration’ hypothesis revealed that once amyloid-β deposition is initiated by independent events, there might be toxic excitation of affected neurons, which induced higher functional connectivity to hasten this deposition. The ‘brain reserve’ hypothesis indicated that enhanced functional connectivity reflects a stable capacity to resist amyloid-β deposition and keep normal cognition. Future longitudinal studies would be needed to explain these possibilities.

As we known, DMN and the ECN were reciprocally anti-correlated networks (Fox et al., 2005; Menon and Uddin, 2010). There might be a dynamic balance between the two insular connecting anticorrelate networks. When the balance was destroyed due to the AD pathology, for example, the ECN disconnection to the insula might promote a heightened connecting DMN to keep the balance, which reflects compensation for the damage of the disrupted network. On the other hand, the aberrant hyperactivity of the DMN induced by the amyloid deposition might cause the disrupted connectivity of ECN.

### Correlation Between RSFCs and Cognitive Performances

The correlation analysis showed that the RSFC between the left dAI and clusters in the thalamus is correlated with MMSE score in the AD patients. This is consistent with the functional role of the ECN, which suggests the frontal-parietal disconnection contribute to the cognitive impairment in AD patients. We also found the disconnection between the dAI and several regions showed negative correlations with cognitive behavior scores, which reflect the implications of these regions for cognitive function.

### Analysis by Removing the Global Signal

The global signal was a time course obtained by averaging resting-state time series over the entire brain (Macey et al., 2004). In RSFC analysis, global signal was commonly removed using a general linear model technique to improve the specificity of functional connectivity analysis and used to map the anti-correlated intrinsic networks (DMN vs. task positive network) in the brain (Fox et al., 2005). The global signal was also believed to afford increased tissue sensitivity (Fox et al., 2009). A recent neurophysiological investigation demonstrated that both positive and negative BOLD correlations have neurophysiological meaning, which reflected fluctuations of spontaneous neuronal activity (Keller et al., 2013). Recent studies found that the insula played a critical role in the dynamic switching between the task positive networks (ECN and SN) and negative network (DMN) (Sridharan et al., 2008; Menon and Uddin, 2010; Liang et al., 2016). Based on the above, our study focused on both the positive and negative correlations between insular subregions and other brain network regions, as well as the changes in the AD patients. To show the relationship among the insular subregions and other brain networks, we analyzed these data and mainly show the result of removing the global signal, but as reference to compare, we also performed the preprocessing again without removing the global signal and the results are shown in the Supplementary Materials.

As we known, the global signals may lead to differences of measured functional connectivities, to explore the problem, we decide to investigate whether the functional results could be influenced by global signals. When comparing the results with or without the global signals, we found the results of positive connectivity were similar, for example, For the left dAI, we observed decreased positive RSFCs mainly in the right IFG, left thalamus, right lingual gyrus, and left MTG in the AD patients without removing the global signal; while by removing global signals, the decreased RSFCs regions were located in the right IFG, left thalamus, and MCC. There was the same situation for the other insular subregions. Together with the previous studies (Macey et al., 2004; Fox et al., 2005, 2009), the results of RSFCs were partly influenced by global signals, but the patterns were approximately consistent between with and without removing global signals. It implies that our results of RSFCs of insular subregions by removing global signals could reflect the changes in intrinsic brain functional activities in the AD patients.

### Future Consideration

There are also several limitations need to be considered in our study. First, in the current study, we only focused on the resting state fMRI data. In the future, we can combine multimodal MRI methods such as fMRI, DTI and perfusion to analyze the brain function, structure and blood flow of the AD patients simultaneously, which may be helpful for deep understanding of the mechanisms of AD. Second, the interference of some potential confounding factors, such as respiratory and cardiac cycle artifacts couldn’t be excluded due to slow sampling rates. Noises from the respiratory and cardiac cycle can interfuse into the resting-state low frequency ranges at slow sampling rates (in this study TR = 2 s). Third, we didn’t gather physiological measurements, gene data or other biological related recordings due to experiment design and equipment restriction. In the future, we will collect the gene, cerebral spinal fluid (CSF), as well as amyloid-β plaques image to acquire more complete experiment data. Fourth, we didn’t acquire the other behavior data such as motor, language, insomnia and so on. In the future we will try our best to gather more behavior data to perform the correlation analysis. Finally, recent studies have focused more on individuals at high risk for AD, in the future, exploring these population might be valuable for the early diagnosis of AD.

## Conclusion

This study showed that different sub-regions of insula can present different connectivity patterns, belonging to distinct functional brain networks such as DMN, ECN, SN, and SMN, which are differently affected in the AD patients.

## Author Contributions

XL, XC, and WZ: the conception or design of the work, the acquisition, analysis, and the interpretation of data for the work, drafting the work, final approval of the version to be published, and agreement to be accountable for all aspects of the work. MX, YiH, HS, and KL: the acquisition of data for the work and clinical evaluation. YoH and ZW: the design of the work, revising the work, final approval of the version to be published, and agreement to be accountable for all aspects of the work.

## Conflict of Interest Statement

The authors declare that the research was conducted in the absence of any commercial or financial relationships that could be construed as a potential conflict of interest.

## Footnotes

1 http://www.fil.ion.ucl.ac.uk/spm/software/spm8/

## Supplementary Material

FIGURE S1 The significant decreased positive RSFCs of bilateral dorsal anterior insula (dAI) in AD patients (*p* < 0.001, uncorrected).

FIGURE S2 The significant decreased positive RSFCs of bilateral dorsal anterior insula (PI) in AD patients (*p* < 0.001, uncorrected).

FIGURE S3 The significant decreased positive RSFCs of left ventral anterior insula (vAI) in AD patients (*p* < 0.001, uncorrected).

TABLE S1 Regions showing decreased positive RSFCs in insular subregions in AD.
