# Supplementary figures and images for: Altered Functional Connectivity of Insular Subregions in Alzheimer’s Disease
Source: Front Aging Neurosci. 2018 Apr 11;10:107. doi: 10.3389/fnagi.2018.00107 (PMC5905235; doi:10.3389/fnagi.2018.00107)

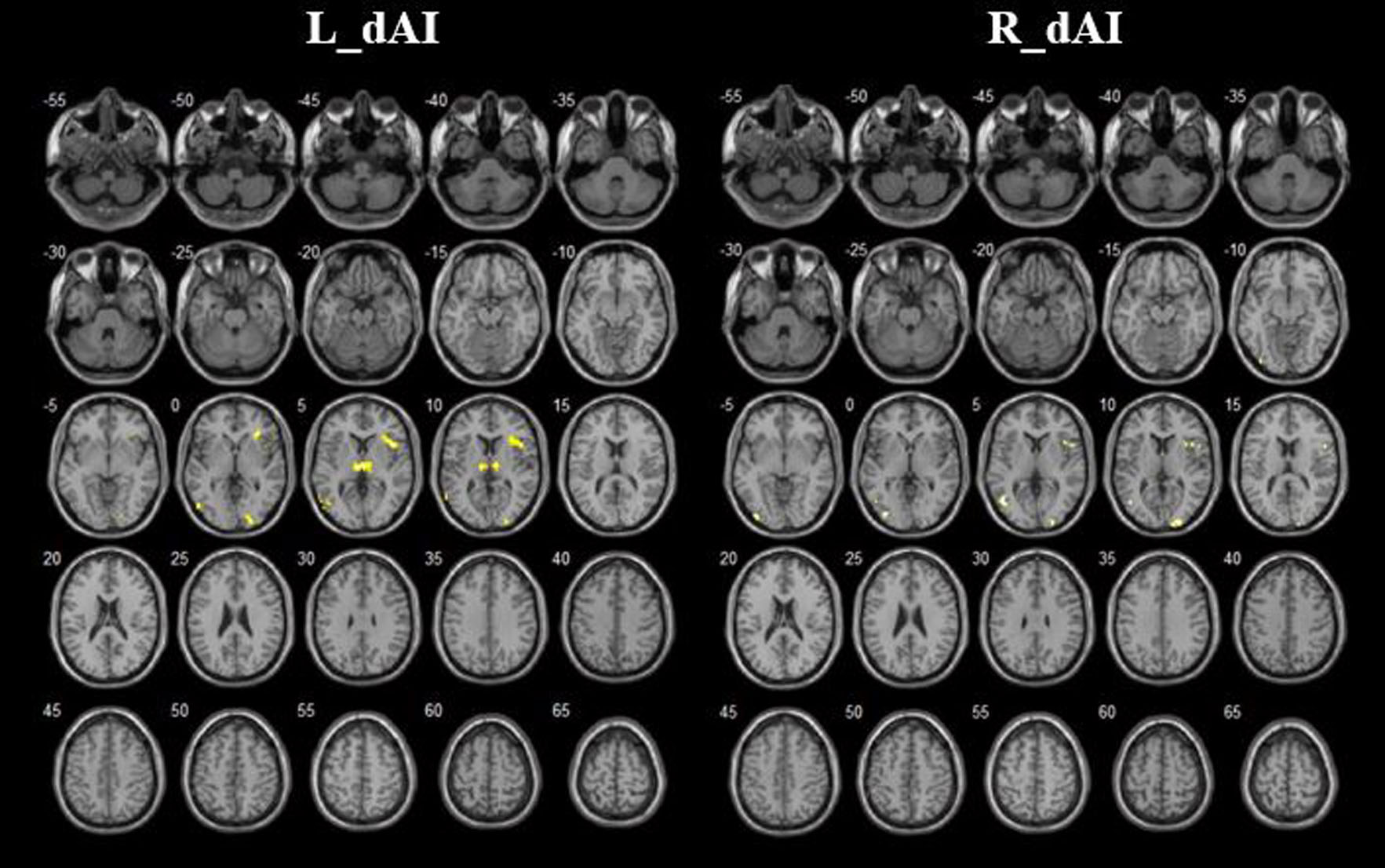

Supplement: FIGURE S1 — The significant decreased positive RSFCs of bilateral dorsal anterior insula (dAI) in AD patients (p < 0.001, uncorrected). [file Image_1.JPEG]

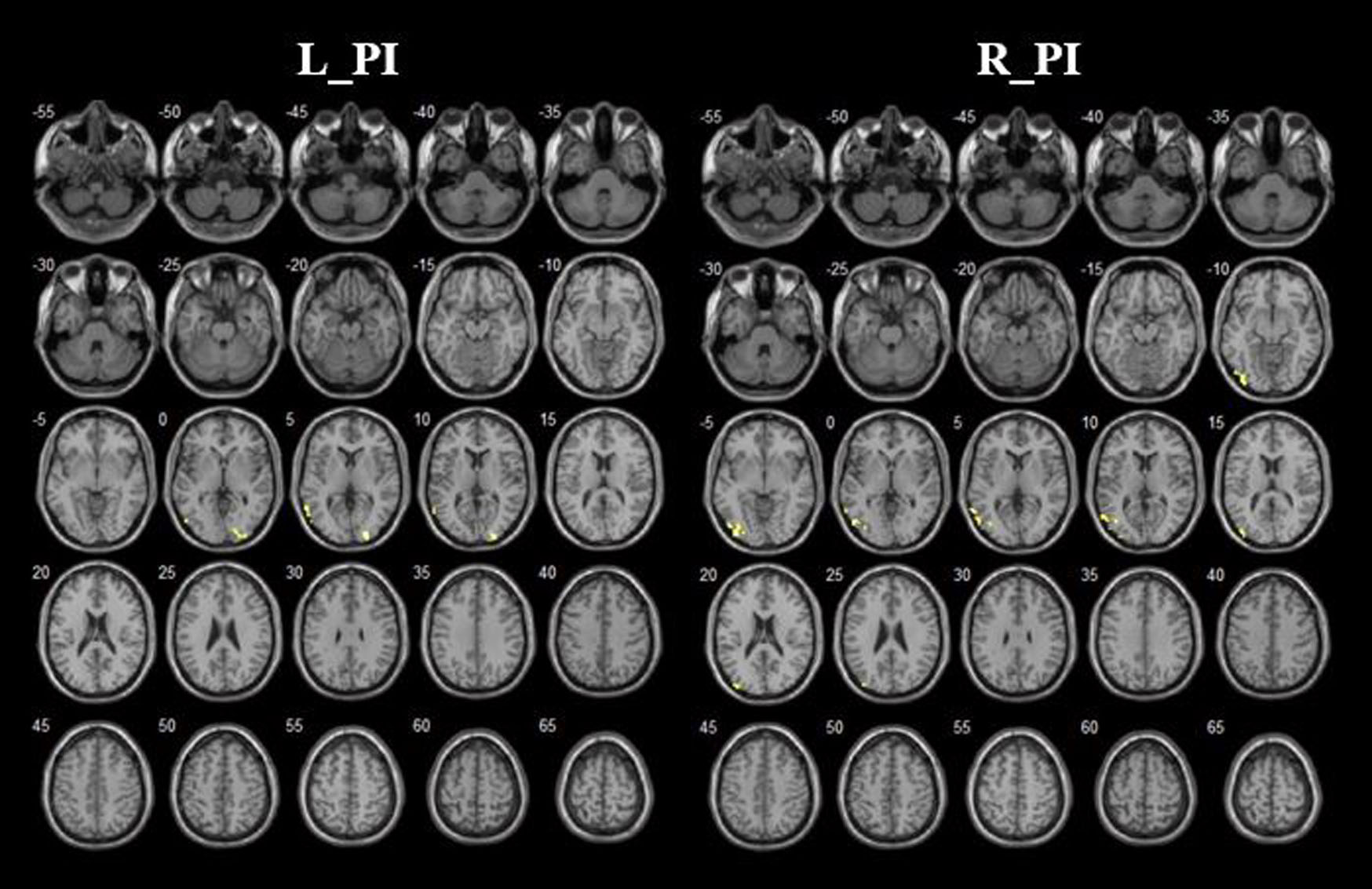

Supplement: FIGURE S2 — The significant decreased positive RSFCs of bilateral dorsal anterior insula (PI) in AD patients (p < 0.001, uncorrected). [file Image_2.JPEG]

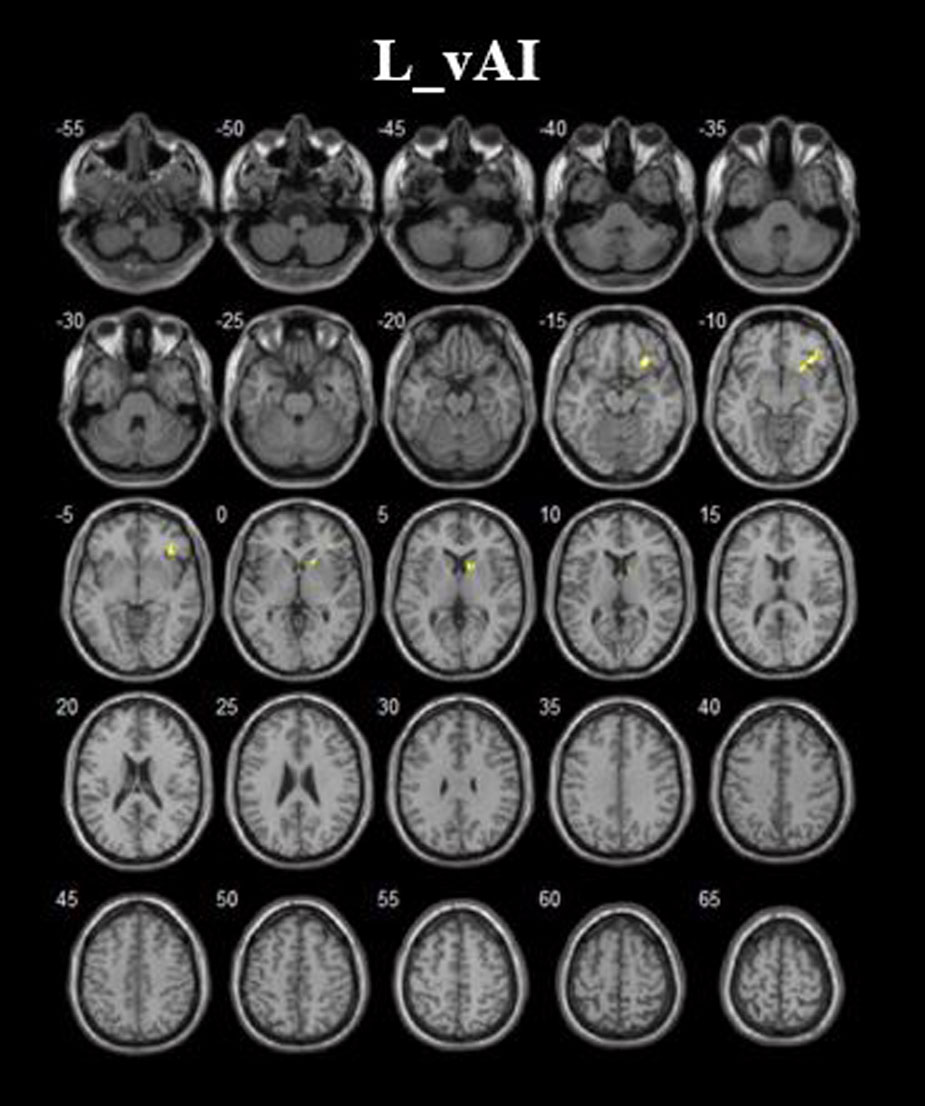

Supplement: FIGURE S3 — The significant decreased positive RSFCs of left ventral anterior insula (vAI) in AD patients (p < 0.001, uncorrected). [file Image_3.JPEG]
