# Supplementary material for: Altered Functional Connectivity of Insular Subregions in Alzheimer’s Disease
Source: Front Aging Neurosci. 2018 Apr 11;10:107. doi: 10.3389/fnagi.2018.00107 (PMC5905235; doi:10.3389/fnagi.2018.00107)
Supplement: TABLE S1 — Regions showing decreased positive RSFCs in insular subregions in AD. [file Table_1.DOCX]

**Supplementary Table. Regions showing decreased positive RSFCs in insular subregions in AD**

| ROIs | Brain regions | Cluster  voxels | MNI  coordinates(mm) | | | Maximum Z |
| --- | --- | --- | --- | --- | --- | --- |
|  |  |  | x | y | z |  |
| L. PI | R. MOG | 48 | 24 | -93 | 6 | -4.53 |
|  | L. MTG | 26 | -63 | -57 | 3 | -4.00 |
| L. dAI | R. IFG | 88 | 42 | 18 | 6 | -5.52 |
|  | R. Lingual | 30 | 21 | -90 | -3 | -4.49 |
|  | L. MTG | 29 | -54 | -75 | 0 | -4.03 |
|  | L. Thalamus | 66 | -3 | -18 | 6 | -5.03 |
| L. vAI | R. IFG | 63 | 36 | 27 | -9 | -5.13 |
|  | R. Caudate | 21 | 12 | 12 | 3 | -4.07 |
| R. PI | L. MOG | 146 | -51 | -78 | -9 | -4.47 |
| R. dAI | L. MOG | 24 | -39 | -87 | -6 | -4.32 |
|  | R. SOG  L. MTG | 30  24 | 24  -45 | -96  -63 | 9  6 | -4.33  -4.23 |
|  | _R. IFG | 29 | 54 | 12 | 12 | -4.10 |

Between groups differences were determined by two-sample t tests (p<0.001, uncorrected)

**Abbreviations:** MOG, middle occipital gyrus; MTG, middle temporal gyrus; IFG, Inferior frontal gyrus; SOG, superior occipital gyrus. L, left; R right.

**Supplementary Figure Legend.** The decreased positive RSFCs of insular subregions in AD patients.
